# Sleep restriction exacerbates cardiac dysfunction in diabetic mice by causing cardiomyocyte death and fibrosis through mitochondrial damage

**DOI:** 10.1038/s41420-024-02214-w

**Published:** 2024-10-21

**Authors:** Jingyi Zhang, Xu Zhao, Jing Tang, Ce Liu, Yining Zhang, Cheng Cai, Qingfeng Du

**Affiliations:** 1 Centre of General Practice, The Seventh Affiliated Hospital of Southern Medical University, Foshan, China; 2 Department of Laboratory Medicine, The Seventh Affiliated Hospital of Southern Medical University, Foshan, China; 3 School of Basic Medical Sciences, Henan University of Science and Technology, Luoyang, China; 4 School of Traditional Chinese Medicine, Southern Medical University, Guangzhou, China; 5 Guangdong Provincial Key Laboratory of Chinese Medicine Pharmaceutics, Guangzhou, China; 6 Hospital of Integrated Traditional Chinese and Western Medicine, Southern Medical University, Guangzhou, China; 7 Guangdong Basic Research Center of Excellence for Integrated Traditional and Western Medicine for Qingzhi Diseases, Guangzhou, China

**Keywords:** Cardiac hypertrophy, Diabetes complications

## Abstract

Diabetic cardiomyopathy (DCM) is a cardiovascular complication of diabetes mellitus with a poor prognosis and is the leading cause of death in diabetic patients. Sleep deficiency is not only recognized as an important risk factor for the development of type 2 DM, but is also associated with increased morbidity and mortality of cardiovascular disease. The underlying role and mechanisms of sleep restriction (SR) in DCM are far from clear. The KK/Upj-Ay mouse model of T2 DM was used as a study subject, and the small animal ultrasound imaging system was used to detect the function of the heart; immunopathological staining was used to clarify the histo-structural pathological alterations of the heart; and TUNEL staining, qPCR, transmission electron microscopy (TEM), and ELISA kits were used to detect apoptosis, oxidative stress, inflammation, and mitochondrial damage, and related molecular alterations. SR led to a significant increase in mortality, cardiac hypertrophy, necrosis, glycogen deposition and fibrosis further deteriorated in DM KK mice. SR increased cardiomyocyte death in KK mice through the Bax/Bcl2 pathway. In addition to this, SR not only exacerbated the inflammatory response, but also aggravated mitochondrial damage and promoted oxidative stress in KK mice through the PRDM16-PGC-1α pathway. Overall, SR exacerbates structural alterations and dysfunction through inflammation, oxidative stress, and apoptosis in DM KK mice, increasing the risk of death. Clinicians and diabetic patients are prompted to pay attention to sleep habits to avoid accelerating the transition of DCM to heart failure and inducing death due to poor sleep habits.

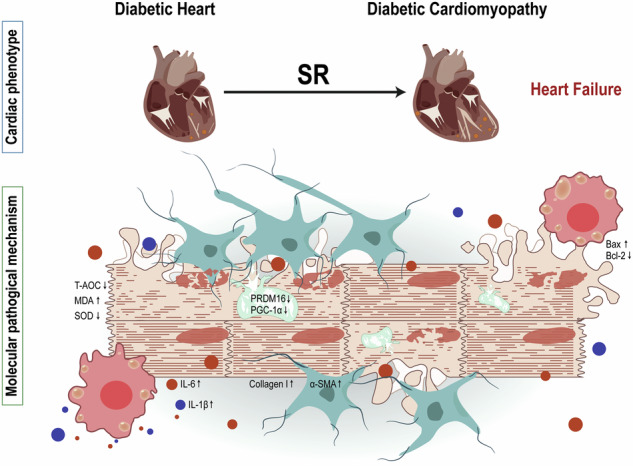

## Introduction

Diabetes is a public health issue of global concern. In 2021, there were approximately 537 million adults with diabetes globally, of which 90–95% will be type 2 diabetes mellitus (T2DM). The global prevalence of diabetes is still rising due to rising obesity rates, changing dietary habits, and sedentary lifestyles, and the number of people with diabetes is expected to increase to 783 million by 2045 [1]. Diabetes is a chronic disease in which long-term disturbed blood glucose and lipids lead to irreversible cardiovascular damage [2, 3]. Diabetic cardiomyopathy (DCM) has a poor prognosis and progresses to heart failure without timely intervention, which is one of the most prominent causes of death in diabetic patients [4, 5]. The major pathological changes in DCM include myocardial fibrosis, cardiac hypertrophy, oxidative stress, and increased inflammatory responses. These structural changes lead to cardiac contractile dysfunction inducing heart failure [4, 6, 7].

Extensive studies have shown that inflammatory response, mitochondrial damage, and fibrosis are hallmarks and major mechanisms in the development of DCM [8]. Elevated inflammation induced by hyperglycemia and oxidative stress caused by mitochondrial damage further promote myocardial fibrotic remodeling and contractile dysfunction [9]. Collagen deposition is a major feature of fibrosis [10]. Increased myocardial fibrosis leads to increased cardiac weight and decreased compliance, impairing the contractile function of the heart leading to heart failure [6]. Insulin resistance, hyperglycemia, lipid metabolism disorders, abdominal obesity and hypercoagulable states present in diabetic patients are risk factors for DCM [4], but nowadays, poor lifestyle such as sleep deprivation due to various factors has become a new risk factor for DCM [11]. Clinical data show that the coexistence of sleep restriction and diabetes are associated with a higher risk of cardiovascular disease, coronary artery disease, stroke and death than sleep restriction or diabetes alone [11]. However, there are no animal studies to confirm this idea.

Adequate sleep, one of the most important forms of health-promoting behavior for humans, is key to ensuring physical and mental health and preventing disease [12–14]. Sleep has long been taken for granted as an integral part of our lives, but in today’s society, a variety of potential and actual pressures have led to widespread sleep procrastination behaviors, manifested in delayed bedtimes and shortened sleep duration, resulting in sleep deprivation [15, 16]. Currently about 27% of the global population suffers from sleep disorders. Sleep deprivation poses a direct and imminent threat to human health, and even a small amount of sleep deprivation can have a significant impact on cardiovascular function. According to studies more than one-third of people with type 2 diabetes tend to have subjective sleep disorders due to fear of poor glycemic control and diabetic complications, making type 2 diabetes often coexist with objective or subjective sleep disorders [17]. Sleep deprivation related to sleep duration and quality is not only recognized as an important risk factor for the development of type 2 diabetes [12], but also, is associated with cardiovascular disease morbidity and mortality [18]. Whether prolonged sleep restriction exacerbates DCM leading to increased mortality and the regulatory mechanisms involved also still require further investigation.

To date, the effects of sleep restriction on DCM have not been investigated, which prompted this study. Therefore, our study explored whether SR exacerbates DCM in KK mice leading to increased mortality and the underlying mechanisms involved.

## Results

### SR caused a significant increase in mortality of KK mice

**Fig. 1:**
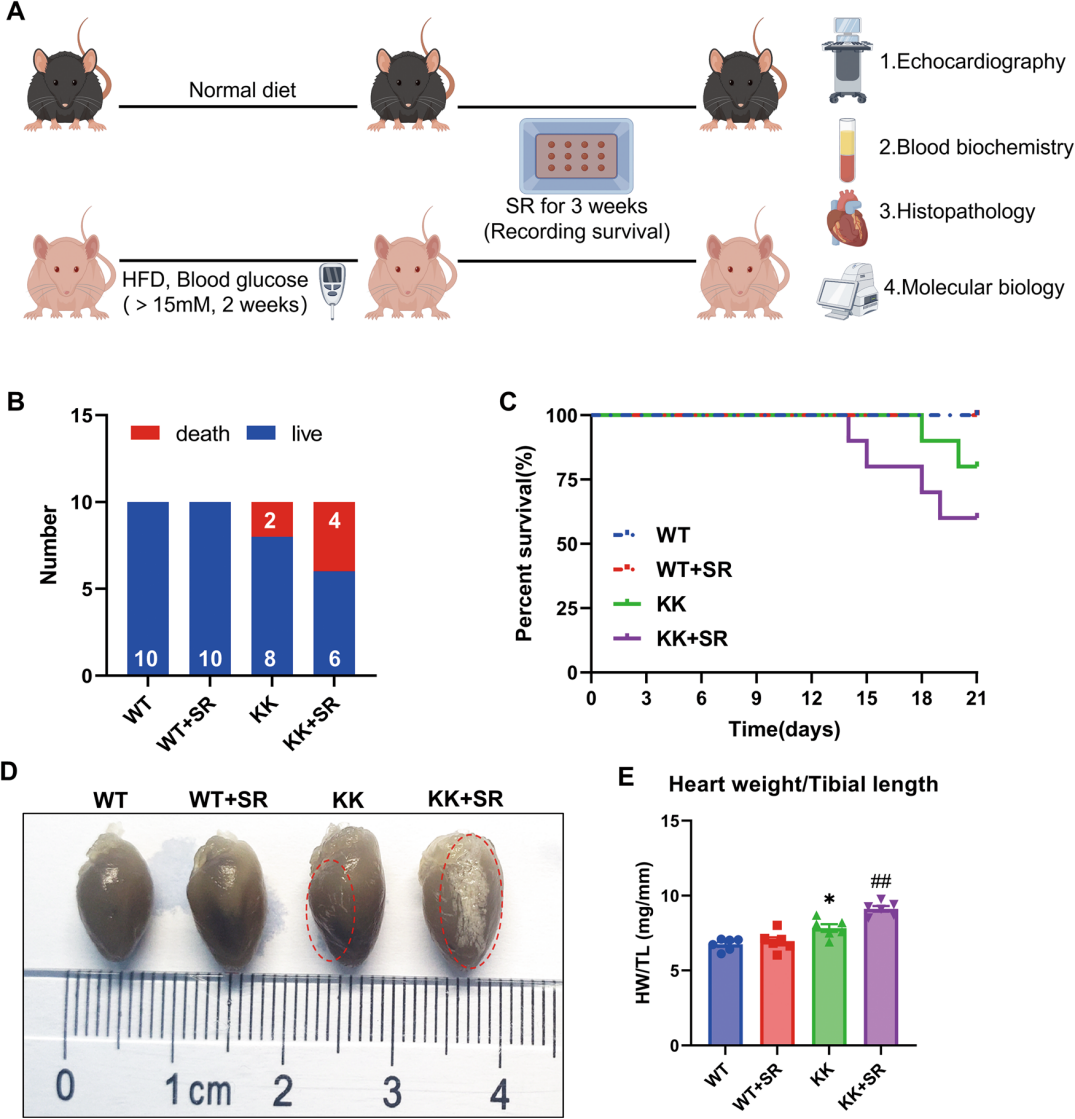
SR causes increased mortality and cardiac hypertrophy in diabetic KK mice. **A** Schematic diagram of the experimental design model. KK mice were given a high-fat chow diet (HFD) and blood glucose greater than 15 mM for two consecutive weeks to establish a T2 DM model, while wild-type C57 BL6/J mice were used as a control group. **B** Statistical plots of the number of mice that survived and died in each group (*n* = 10 per group). **C** The effects of SR on the mortality of KK mice using the Kaplan–Meier method to measure survival rates (*n* = 10 per group). **D** Representative images of the heart. **E** Heart weight/tibial length ratio (HW/TL) showing the relative heart weight (*n* = 6 per group). The data are presented as the mean ± SEM. Differences between groups were assessed with Two-way ANOVA followed by Bonferroni’s *post hoc* test. *p < 0.05 versus the WT group; ^##^*p* < 0.01 versus the KK group.

DCM is one of the serious complications of diabetes. Clinical data suggest that the coexistence of sleep restriction and diabetes mellitus is associated with a higher risk of cardiovascular disease, coronary artery disease, stroke and death than sleep restriction or diabetes mellitus alone. However, there is no animal experiment to confirm this idea. KK mouse is the classic diabetic mouse model, and we next examined the survival rate of KK mice to investigate the effect of SR on mortality. The results showed that the mortality rate of KK mice was 20% at 21 days and increased to 40% after SR (Fig. 1B, C). To investigate the cause of death, we performed autopsies at the end of the experiment. We found striated white lesions on the cardiac surface of KK mice, whereas SR + KK mice had more extensive lamellar white lesions on the cardiac surface (Fig. 1D). The heart weight/tibia length ratio was significantly increased in KK mice compared with WT mice, while SR exacerbated the increase in heart weight/tibia length ratio in KK mice (Fig. 1E). Taken together, we suggest that SR causes increased mortality in KK mice that may be associated with cardiac damage.

### SR exacerbates cardiac systolic dysfunction in diabetic KK mice, accompanied by histopathological changes

**Fig. 2:**
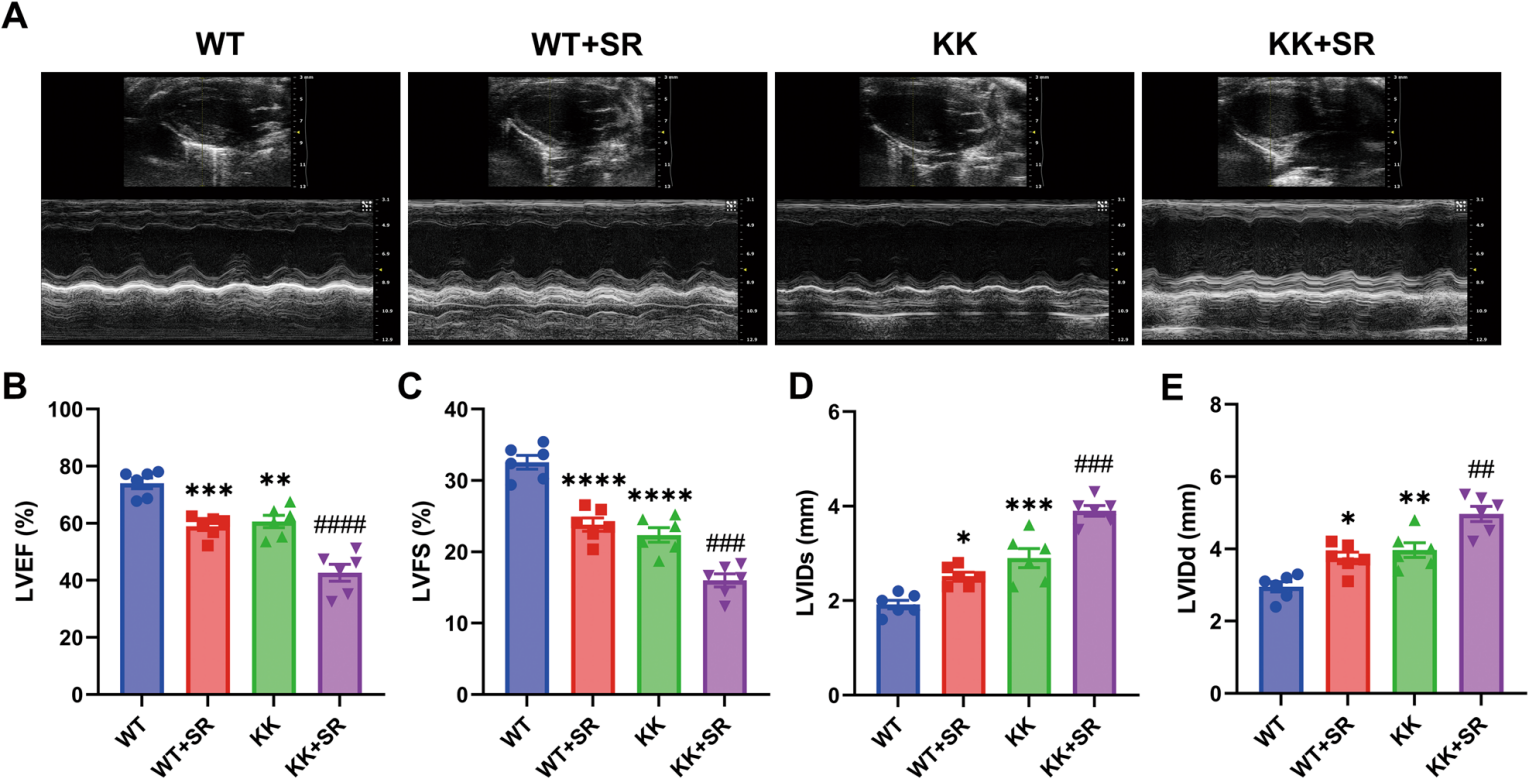
SR aggravates cardiac dysfunction caused by DM. **A** Representative echocardiographic images showing the effects of SR on cardiac function in WT and diabetic KK mice. **B**–**D** The percentage of left ventricular ejection fraction (%LVEF) and fractional shortening (%LVFS), left ventricular systolic internal dimension (LVIDs) and left ventricular diastolic internal dimension (LVIDd) were quantified (*n* = 6 per group). The data are presented as the mean ± SEM. Differences between groups were assessed with Two-way ANOVA followed by Bonferroni’s *post hoc* test. *p < 0.05, **p < 0.01, ***p < 0.001, ****p < 0.0001 versus the WT group; ^##^*p* < 0.01, ^###^*p* < 0.001, ^####^*p* < 0.0001 versus the KK group.

**Fig. 3:**
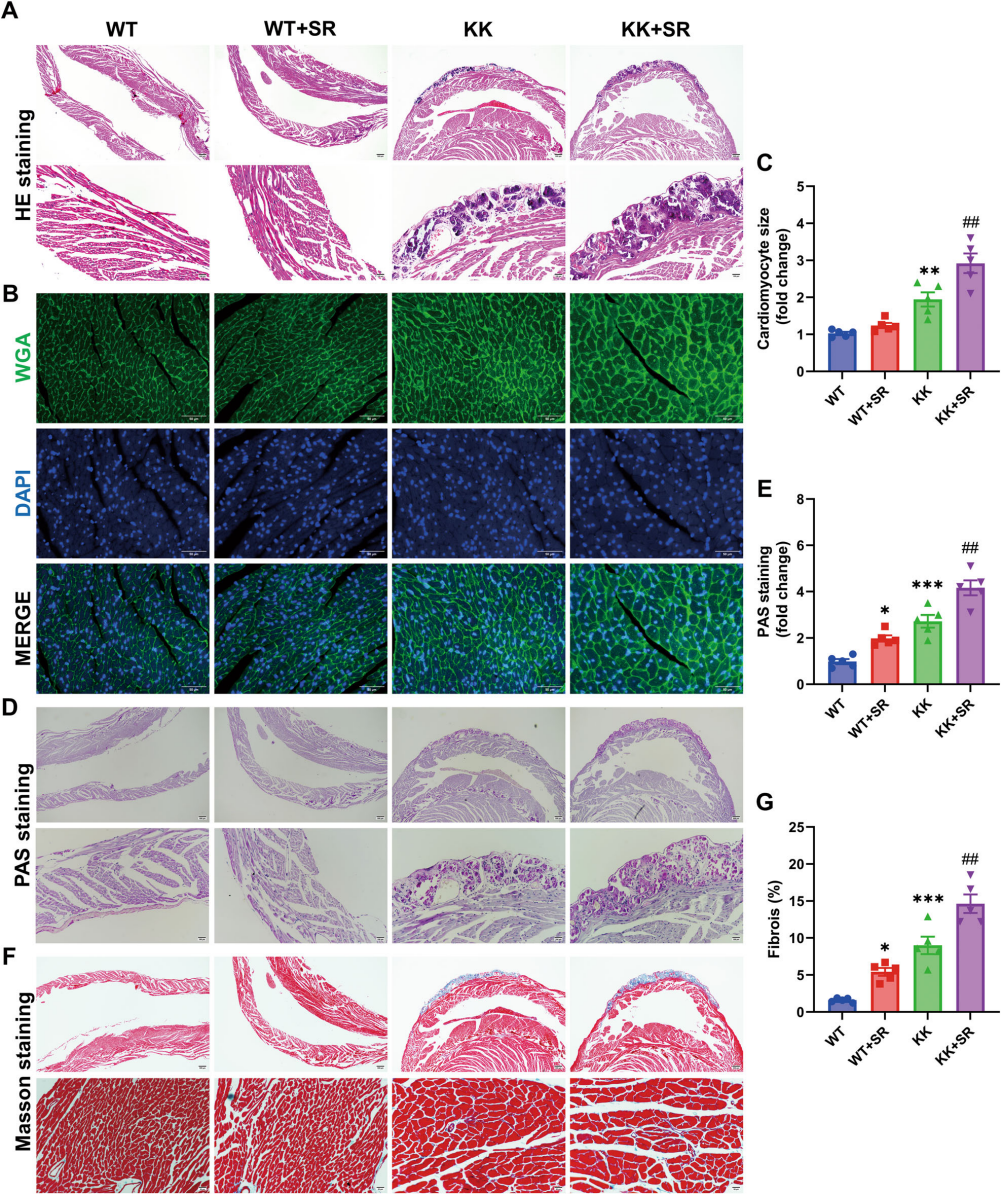
SR exacerbates cardiomyocyte hypertrophy, glycogen deposition and fibrosis in DM KK mice. **A** Representative photographs of the myocardium with H&E staining. Scale bar: 100 μm. **B** Representative photographs of the myocardium with WGA staining. Scale bar: 50 μm. **C** Relative cardiomyocyte size in myocardial tissue (*n* = 5 per group). **D** Representative photographs of the myocardium with PSA staining. Scale bar: 500 μm and 100 μm. **E** Relative value of PSA staining-positive area in myocardial tissue (*n* = 5 per group). **F** Representative photographs of the myocardium with Masson staining. Scale bar: 500 μm and 100 μm. **G** Percentage of fibrotic area in myocardial tissue (*n* = 5 per group). The data are presented as the mean ± SEM. Differences between groups were assessed with Two-way ANOVA followed by Bonferroni’s *post hoc* test. *p < 0.05, **p < 0.01, **p < 0.001 versus the WT group; ^##^*p* < 0.01 versus the KK group.

To investigate the effects of SR on cardiac function in KK mice, we used an ultrahigh-frequency high-resolution small animal ultrasound imaging system to continuously acquire multiple left ventricular ultrasound images for measurement and analysis (Fig. 2A). The results showed that the percentage of left ventricular ejection fraction (%LVEF) and the percentage of left ventricular fractional shortening (%LVFS) were decreased in KK mice compared with WT mice (Fig. 2B, C). The left ventricular systolic internal dimension (LVIDs) and left ventricular diastolic internal dimension (LVIDd) were significantly increased in KK mice (Fig. 2D, E). SR exacerbated these changes in KK mice. To evaluate cardiac injury, histopathological staining was performed to detect histopathological changes in the myocardium. HE staining showed increased subepicardial necrotic myocardium in KK mice (Fig. 3A). Wheat germ agglutinin (WGA) staining was used to measure cardiomyocyte size, and the results showed a significant increase in cardiomyocyte size in the KK mice compared with the WT group (Fig. 3B, C). PAS staining and Masson staining showed increased glycogen deposition and fibrosis in the myocardium of KK mice (Fig. 3D–G). SR exacerbates these pathologic changes in the hearts of KK mice. These results suggest that SR exacerbated cardiac dysfunction, cardiomyocyte hypertrophy, necrosis, glycogen deposition and fibrosis in KK mice.

### Effect of SR on hypertrophy markers and fibrosis-related parameters in myocardial tissue

**Fig. 4:**
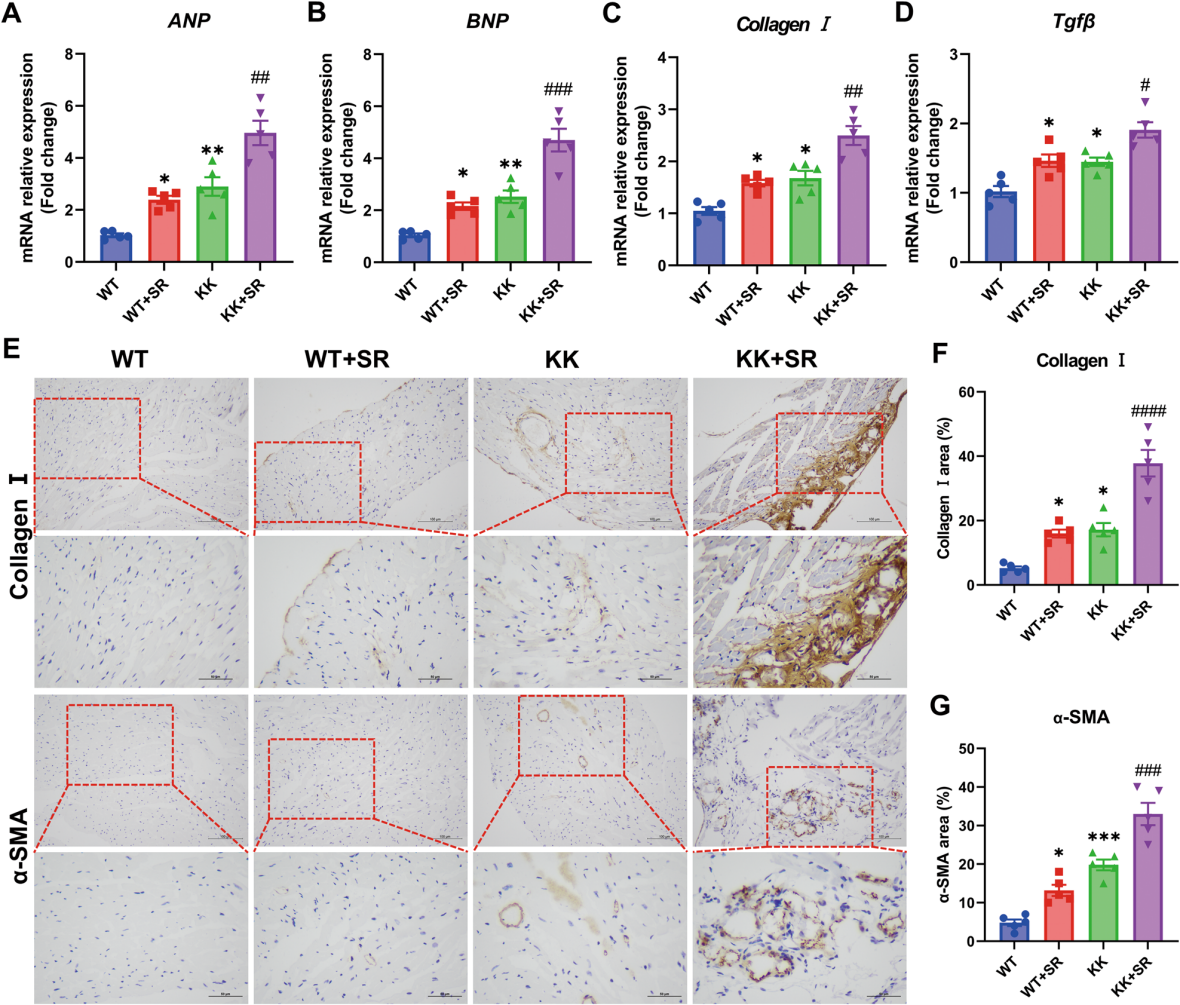
SR leads to a further increase in the expression levels of hypertrophy- and fibrosis-related molecules in myocardial tissues of KK mice. **A**, **B** Relative mRNA levels of *ANP* and *BNP* (*n* = 5). **C**, **D** Relative mRNA levels of *Collagen I* and *Tgfb* (*n* = 5). **E** Representative photographs of immunohistochemical staining for Collagen I and α-SMA in myocardium. Scale bar: 100 μm and 50 μm. **F** Percentage of Collagen I -positive area in myocardial tissue (*n* = 5 per group). **G** Percentage of α-SMA-positive area in myocardial tissue (*n* = 5 per group). The data are presented as the mean ± SEM. Differences between groups were assessed with Two-way ANOVA followed by Bonferroni’s *post hoc* test. *p < 0.05, **p < 0.01, ***p < 0.001 versus the WT group; ^#^*p* < 0.05, ^##^*p* < 0.01, ^###^*p* < 0.001, ^####^*p* < 0.0001 versus the KK group.

Next, we examined the expression changes of hypertrophic markers and fibrosis-related parameters. qPCR results showed elevated expression levels of hypertrophic markers (*ANP* and *BNP*) and fibrosis-related parameters (*Collagen I* and *Tgfβ*) in KK mice (Fig. 4A–D). The expression levels of these indicators were further raised in KK mice subjected to SR. In addition, immunohistochemical analysis of the expression levels of collagen I and α-SMA proteins revealed significant differences among the groups. The positive percentages of collagen I and α-SMA increased dramatically in the KK + SR group as compared with the KK group (Fig. 4E–G). These data suggest that SR exacerbates the increase in hypertrophic markers and fibrosis-related parameters in myocardial tissue of diabetic mice.

### SR increases cardiomyocyte death in KK mice through the Bax/Bcl2 pathway

**Fig. 5:**
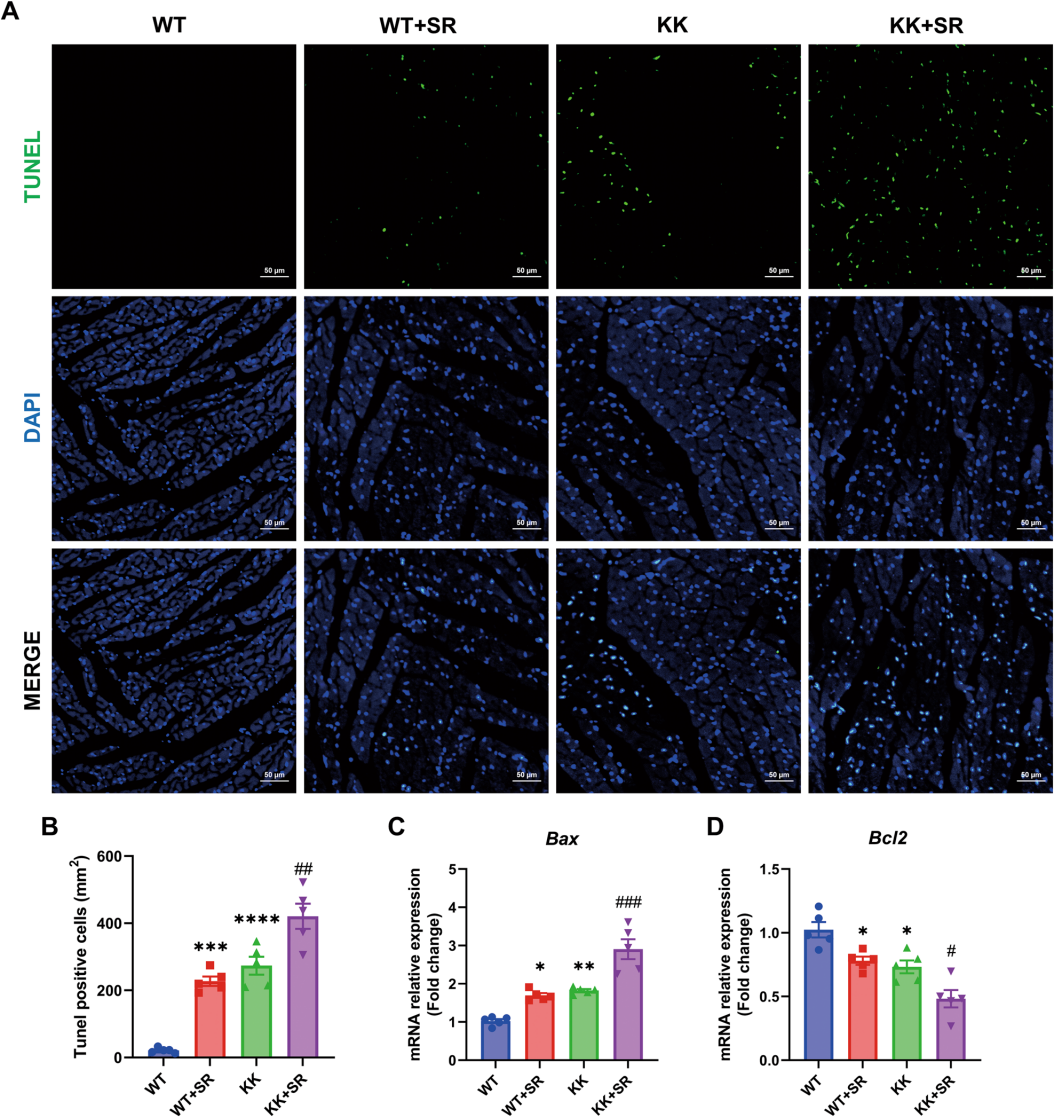
SR increases cardiomyocyte apoptosis in KK mice. **A** Representative photographs of TUNEL staining in myocardium. Scale bar: 50 μm. **B** Statistical plot of the number of TUNEL-positive cells per unit area (*n* = 5 per group). **C**, **D** Relative mRNA levels of *Bax* and *Bcl2* (*n* = 5). The data are presented as the mean ± SEM. Differences between groups were assessed with Two-way ANOVA followed by Bonferroni’s *post hoc* test. *p < 0.05, **p < 0.01, ***p < 0.001, ****p < 0.0001 versus the WT group; ^#^*p* < 0.05, ^##^*p* < 0.01, ^###^*p* < 0.001 versus the KK group.

We used TUNEL staining to detect the effect of SR on cardiomyocyte apoptosis in diabetic KK mice. The results showed that the number of TUNEL-positive cells in the myocardium of KK mice was significantly increased compared with that of WT mice. Meanwhile, the number of TUNEL-positive cells in the KK + SR group was significantly more than that in the KK group (Fig. 5A, B). Next, we used qPCR to detect the expression levels of Bax and Bcl2 in the myocardial tissues of mice in each group. The results showed that the expression of Bax in the myocardium of KK mice was elevated and the expression of Bcl2 was decreased, and this change was more significant in the KK + SR group (Fig. 5C, D). These results suggest that there is cardiomyocyte death in diabetic KK mice and that SR exacerbates KK cardiomyocyte death via the Bax/Bcl2 pathway.

### SR aggravates mitochondria damage in KK mice through the PRDM16-PGC-1α pathway

**Fig. 6:**
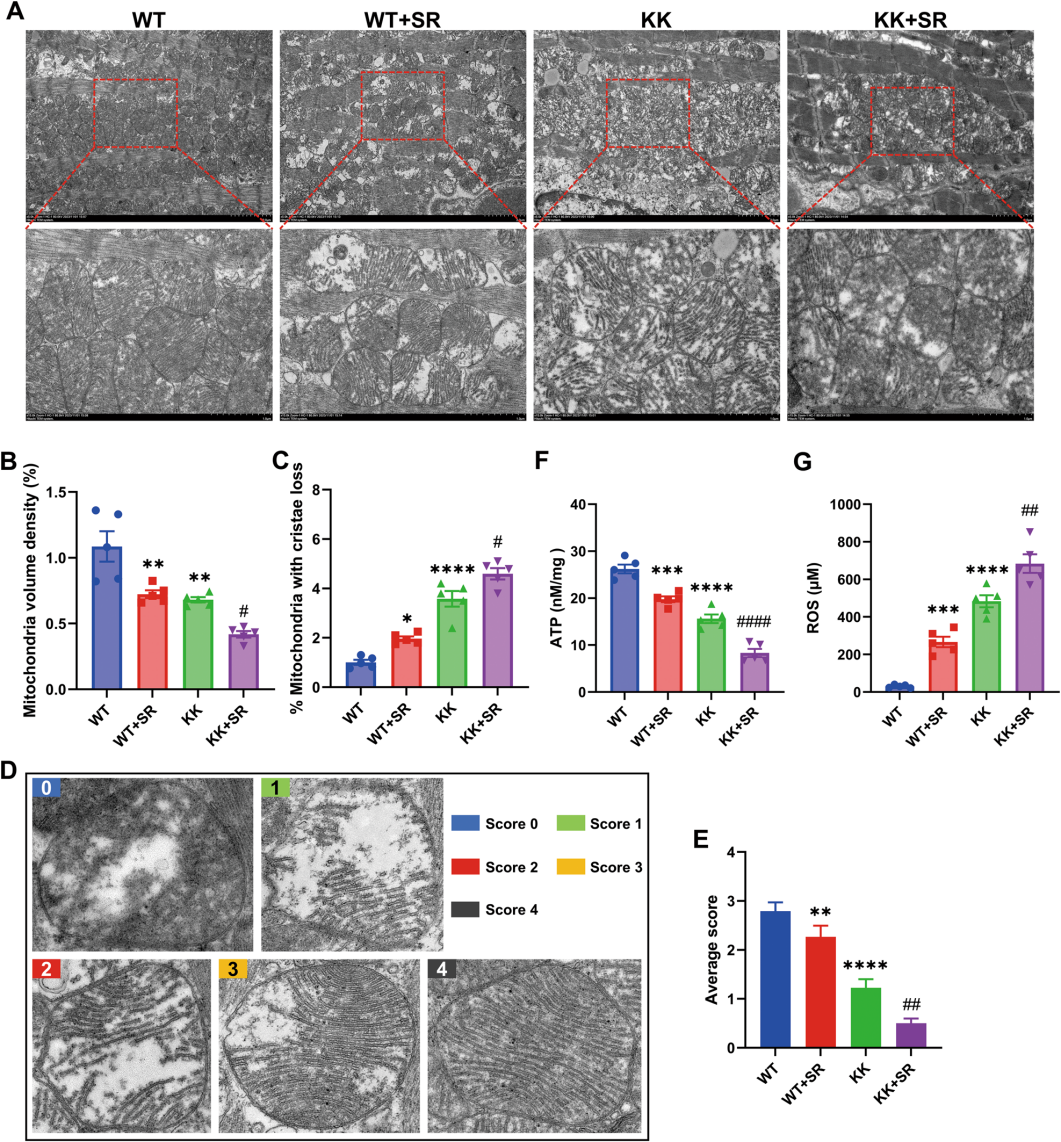
SR aggravates the mitochondria damage in KK mice. **A** Representative images of heart sections from electron microcopy (upper panels, 5000×). Representative EM images showing disarrayed cristae of the matrix in the mitochondria of hearts (lower panels - high magnification of boxed regions in the upper panels, 15,000×). **B** Mitochondria density was determined by the percentage of mitochondria area per field area (10–15 random fields (5000×) per heart, *n* = 5). **C** Quantification of the percent of damaged mitochondria (10–15 random fields (15,000×) per heart, *n* = 5). **D** Representative mitochondrial appearance in EM using a 5-grade scoring system. Score 4, cristae content >80%, well-defined and intact cristae; score 3, cristae content 60–80%, slightly irregular cristae; score 2, cristae content 30–60%, major distortions and discontinuous membranes and cristae; score 1, cristae content 10–30%, severely fragmented or swollen cristae and warped membranes; score 0, cristae content <10%, severely broken membranes with almost absent cristae. **E** Statistical plot showing the overall mean score. A total of 150 mitochondria were randomly analyzed in each sample. **F** ATP content in myocardial tissues of mice in each group (*n* = 5 per group). **G** ROS content in myocardial tissues of mice in each group (*n* = 5 per group). The data are presented as the mean ± SEM. Differences between groups were assessed with Two-way ANOVA followed by Bonferroni’s *post hoc* test. *p < 0.05, **p < 0.01, ***p < 0.001, ****p < 0.0001 versus the WT group; ^#^*p* < 0.05, ^##^*p* < 0.01, ^###^*p* < 0.001 versus the KK group.

We used transmission electron microscopy (TEM) to analyze mitochondrial morphology and function and the results showed that SR exacerbated the reduction of mitochondrial density in the hearts of diabetic KK mice (Fig. 6A, B). Concurrently, SR exacerbated DCM-induced disarrayed cristae (Fig. 6A, C). Further high-resolution electron microscopy analysis of mitochondrial morphology using a modified 5-grade scoring system [19, 20] showed that mitochondrial morphology was severely impaired in diabetic KK mice, including inflation, warped membranes, irregularities, and absence of cristae (Fig. 6D, E). At the same time, ATP concentration was reduced and ROS was increased in myocardial tissues of KK mice, and SR exacerbated these alterations (Fig. 6F, G). These results suggest that SR exacerbates mitochondrial dysfunction in cardiac tissues of KK mice.

**Fig. 7:**
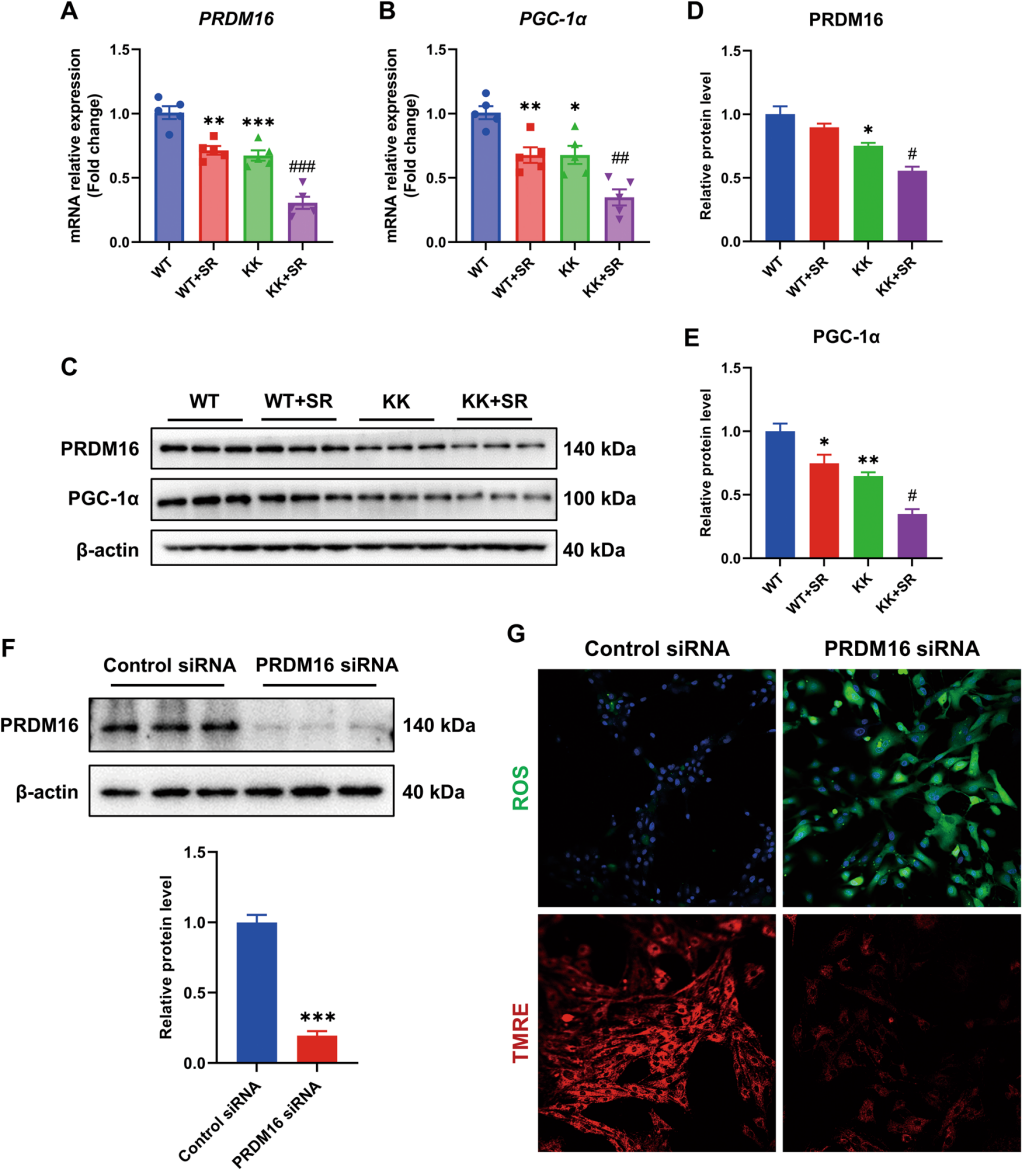
SR exacerbates mitochondrial dysfunction via PRDM16. **A**, **B** Relative mRNA levels of *PRDM16* and *PGC-1α* (*n* = 5). **C**–**F** Western blotting and quantitative analyses were performed to determine PRDM16 and PGC-1α protein expression. β-actin was used as a loading control. **G** Representative images of ROS and TMRE assays of H9C2 cells. Student’s *t*-test was used to compare two groups. The data are presented as the mean ± SEM. Differences between groups were assessed with Two-way ANOVA followed by Bonferroni’s *post hoc* test. *p < 0.05, **p < 0.01, ***p < 0.001, ****p < 0.0001 versus the WT group; ^#^*p* < 0.05, ^##^*p* < 0.01, ^###^*p* < 0.001 versus the KK group. ***p < 0.001 versus the Control siRNA group.

SR significantly exacerbated mitochondrial damage in KK mice. In parallel, we found that the expression of PRDM 16, a novel cardiac regulator of metabolism and energetics in cardiomyopathy, and the downstream target gene, PGC-1α, was decreased in KK mice, and further decreased in the SR + KK group (Fig. 7A–E). These data suggest that SR might exacerbates myocardial mitochondrial damage in diabetic mice by inhibiting PRDM16/PGC-1α. In addition to this, we found that knockdown of PRDM16 expression resulted in elevated cellular ROS and decreased mitochondrial membrane potential (Fig. 7F–G), suggesting a regulatory role of PRDM16 in mitochondrial function in cardiomyocytes.

### SR exacerbates oxidative stress and inflammatory factor expression levels in KK mice

**Fig. 8:**
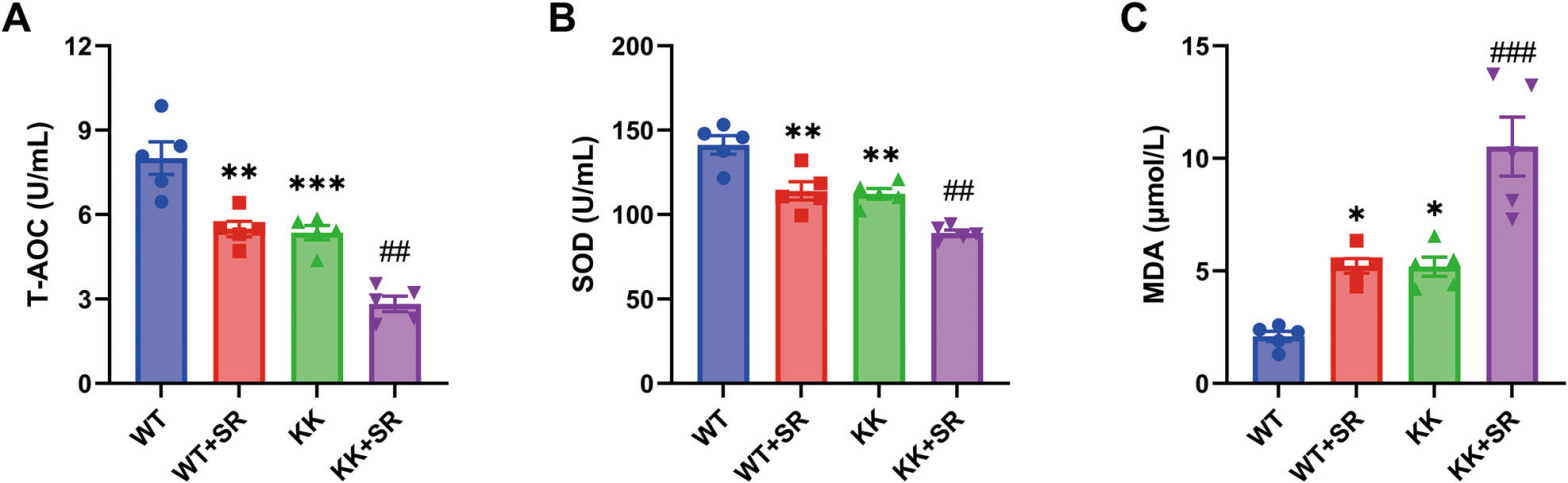
SR promotes oxidative stress in KK mice. The levels of T-AOC (**A**), SOD (**B**) and MDA (**C**) in mouse plasma (*n* = 5 per group). The data are presented as the mean ± SEM. Differences between groups were assessed with Two-way ANOVA followed by Bonferroni’s *post hoc* test. *p < 0.05, **p < 0.01, ***p < 0.001 versus the WT group; ^##^*p* < 0.01, ^###^*p* < 0.001 versus the KK group.

**Fig. 9:**
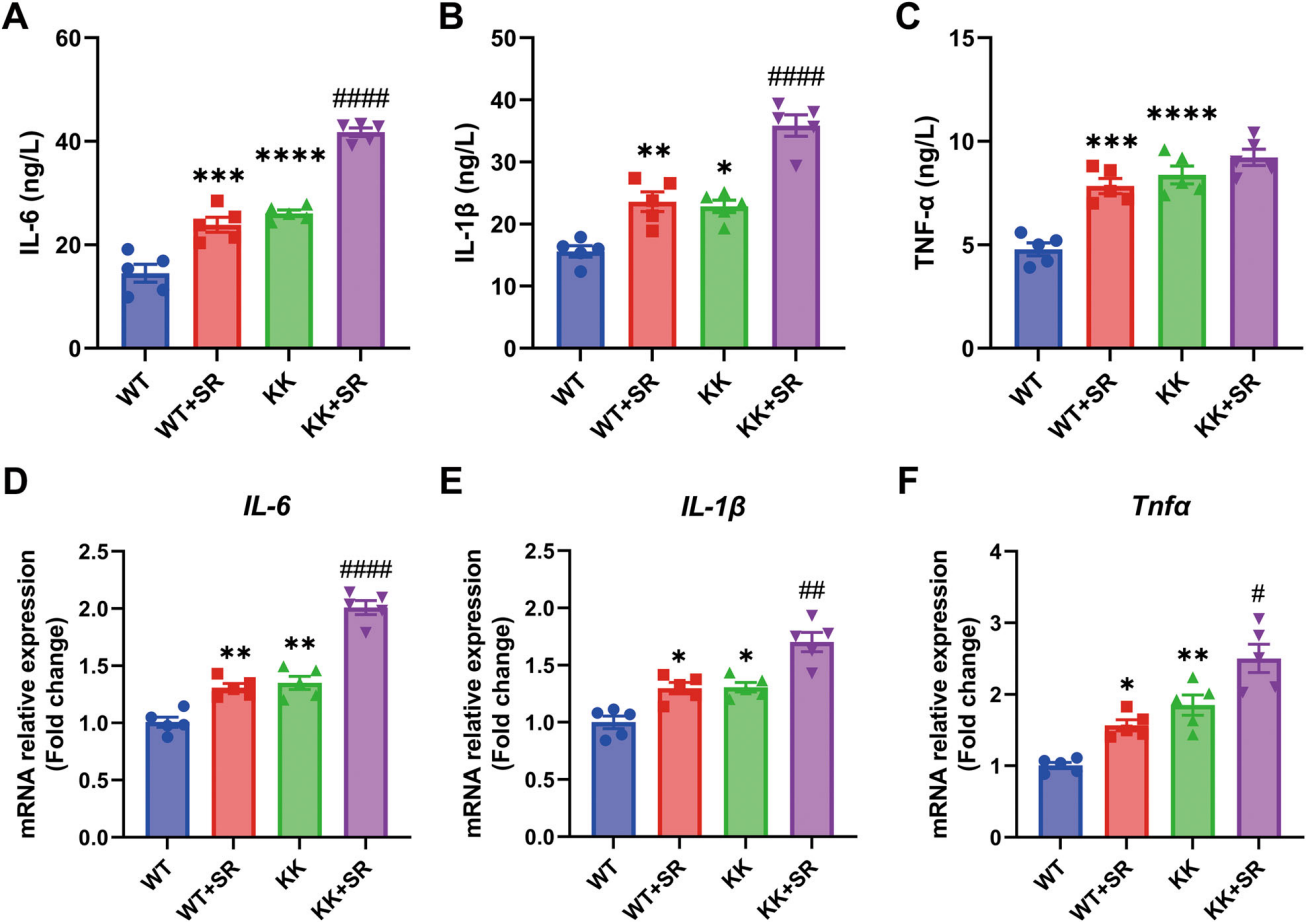
SR exacerbates the inflammatory response in diabetic KK mice. **A**–**C** The levels of IL-6, IL-1β and TNF-α in mouse plasma (*n* = 5 per group). **D**–**F** Relative mRNA levels of *IL-6*, *IL-1β* and *Tnfα* (*n* = 5). The data are presented as the mean ± SEM. Differences between groups were assessed with Two-way ANOVA followed by Bonferroni’s *post hoc* test. *p < 0.05, **p < 0.01, ***p < 0.001, ****p < 0.0001 versus the WT group; ^#^*p* < 0.05, ^##^*p* < 0.01, ^####^*p* < 0.0001 versus the KK group.

Excessive oxidative stress is the causative agent of diabetic cardiomyopathy in mice in response to high blood glucose levels. To determine the effect of SR on oxidative stress in diabetic mice, we measured plasma levels of T-AOC, SOD, and MDA. The results showed that the levels of T-AOC and SOD were decreased and the level of MDA was increased in the KK group compared with the WT group. The levels of T-AOC and SOD in the SR + KK group were lower than those in the KK group, and the level of MDA was higher than that in the KK group (Fig. 8A–C). Chronic inflammation is also a cause of induced cardiomyocyte injury. We found that the expression levels of plasma pro-inflammatory factors IL-6, IL-1β and TNF-α were elevated in the KK group (Fig. 9A, B). In addition to this, the mRNA expression levels of *IL-6*, *IL-1β* and *Tnfα* were also elevated in myocardial tissues of the KK group (Fig. 9C–E). The elevation of pro-inflammatory factor expression levels in plasma and myocardial tissues were more significant in the SR + KK group compared to the KK group. The results indicate that SR can lead to excessive oxidative stress by increasing the levels of oxidative products and decreasing antioxidant enzymes in cardiac tissues of KK mice. Meanwhile, SR promoted the expression of proinflammatory factors and exacerbated the inflammatory response in KK mice.

## Discussion

To date, emerging evidence has emphasized the critical role of inflammatory responses, oxidative stress, and cardiac fibrosis in the development of DCM. SR is a poor lifestyle that promotes oxidative stress while keeping the organism in a prolonged state of chronic inflammation. Consequently, this study investigated the potential of SR to exacerbate DCM in KK mice and elucidated the underlying mechanisms involved. These results suggest that SR promoted DM-induced cardiac dysfunction and hypertrophy; exacerbated mitochondrial damage through inhibition of the PRDM16/PGC-1α pathway; and exacerbated cardiac fibrotic remodeling and cardiac dysfunction through promotion of oxidative stress and inflammatory responses, which ultimately induced increased mortality.

With the upgrading of internet and electronic products, people are constantly compressing their sleep time, and sleep deprivation has become a serious social problem [15, 16]. Sleep deprivation leads to an increase in the incidence of DM [12], but the effect on the cardiovascular complications of DM is not clear. DCM is one of the cardiovascular complications of diabetes, which mainly manifests as cardiomyocyte apoptosis, hypertrophy, fibrosis as well as cardiac systolic and diastolic dysfunction, and is the main cause of heart failure and sudden death in diabetic patients [2–4]. Studies have shown that the risk of cardiovascular disease in diabetic patients is 2–3 times higher than that in normal individuals [21]. DM is an independent risk factor for left ventricular hypertrophy and congestive heart failure, leading to structural changes in the heart that increase susceptibility to cardiovascular disease and worsen the prognosis of patients [22]. Myocardial structural abnormalities in DCM are mainly characterized by myocardial hypertrophy and fibrosis, and these changes amplify LV systolic dysfunction in patients with DM in response to other risk factors, and eventually lead to heart failure and death [2, 4, 8]. In addition to this, clinical studies have shown that short sleep duration is associated with an increased risk of developing or dying from coronary heart disease [23]. Patients with sleep disorders combined with DM have a higher risk of cardiovascular disease, coronary heart disease, stroke and mortality than those with sleep disorders or DM alone [11, 24]. This suggests that sleep deficiency is an important factor in exacerbating the transition from DCM to heart failure, but there are no experimental data to confirm this; therefore, we used the classical DM KK mouse animal model with sleep restriction by the multiplatform water environment method and found that SR resulted in increased mortality in DM KK mice. Gross and histopathological staining of DM KK mouse hearts showed cardiac hypertrophy and fibrosis, which were more pronounced in KK mice after experiencing SR. Significant structural changes lead to functional impairment, and we found that SR exacerbated cardiac systolic dysfunction in KK mice. In conclusion, these results corroborate with the clinical findings [11, 24], and at the same time, the animal model facilitates the collection of cardiac tissues and provides sufficient samples for the next investigation of molecular pathological mechanisms.

Myocardial fibrosis is an important alteration of DCM, and collagen deposition is a key marker of fibrosis [3, 10]. Cardiac fibrosis is mainly mediated by cardiac fibroblasts, which are abundant in the myocardium and usually in an inactive state [6]. After cardiac injury, cardiac fibroblasts differentiate into contractile myofibroblasts [25]. These transformed cells with a myofibroblast phenotype and myofibroblasts are characterized by the expression of contractile proteins such as a-SMA and large collagen deposits [6, 10, 26]. Collagen deposition leads to reduced ventricular compliance, which in turn impairs normal diastolic and systolic function of the heart and ultimately leads to heart failure. In our study, we observed excessive deposition of type I collagen in myocardial tissues of diabetic mice, and SR resulted in further exacerbation of this alteration. In addition, we noted a massive activation of a-SMA+ myofibroblasts consistent with elevated TGF-β expression in cardiac tissues of diabetic mice, and these changes were further worsened after experiencing SR.

More and more studies have shown that the inflammatory response, cardiac hypertrophy, and activation of apoptotic and fibrotic pathways play an important role in the progression of biochemical and pathological changes associated with DCM [27–30]. DCM is characterized by elevated levels of serum markers of cellular injury, increased cardiac hypertrophy, fibrosis, and cell death, accompanied by upregulation of pro-inflammatory factors in diabetic mouse model [6, 31]. Similar results were observed in high glucose-stimulated H9C2 cells [31]. Meanwhile, SR has also been reported to trigger systemic inflammatory state [32, 33], oxidative stress [34], and apoptotic pathways [35], so, we suggest that SR exacerbated KK mice DCM alterations may be associated with inflammation, oxidative stress, and apoptosis. Our results showed that pro-inflammatory factors (IL-6, IL-1β, and TNF-α), oxidative stress products MDA, and the number of apoptosis-positive cardiomyocytes were further elevated, and indices of antioxidant (T-AOC and SOD) were further decreased in KK mice experiencing SR. Thus, increased inflammatory response, oxidative stress and apoptosis expedited the structural and functional deterioration of the DM heart.

Mitochondrial biogenesis plays an important role in maintaining mitochondrial mass and functional homeostasis, and its dysfunction is strongly associated with the development of cardiovascular disease, cancer, and neurodegenerative diseases [36–38]. PRDM16, a transcription factor with a PR structural domain and histone lysine methyltransferase activity [39, 40], has been reported to affect mitochondrial function in both senescent and DM mice [38, 41]. Cardiac-specific defects in PRDM16 accelerate cardiomyopathy and cardiac insufficiency and exacerbate mitochondrial dysfunction and apoptosis in DM mice [38]. Peroxisome proliferator-activated receptor gamma coactivator-1α (PGC-1α) regulates mitochondrial biosynthesis and has been found to be decreased in T2 DM [42, 43], and is a direct target of PRDM16 action [38]. Consistent with these studies, our results show reduced expression of PRDM16 and PGC-1α in the hearts of DM KK mice. In addition to this, we found that SR not only decreases the expression of PRDM16 and PGC-1α in WT mice, but further leads to their low expression in KK mice.

## Conclusion

In summary, SR exacerbates the inflammatory response in diabetic KK mice, exacerbates cardiomyocyte mitochondrial damage and promotes oxidative stress via the PRDM16-PGC-1α pathway, and these increase cardiomyocyte apoptosis via the Bax/Bcl2 pathway. Because of cardiomyocyte death, the heart undergoes adaptive changes with increased fibrotic remodeling, which leads to decreased cardiac compliance, worsening of cardiac contractile dysfunction, accelerated transition to heart failure, and ultimately increased mortality.

### Limitations

There are also many limitations to our study. In addition, the T2 DM model used in our study was based on KK mice, and whether SR plays the same role in T1 DM and other types of T2 DM remains to be investigated. Secondly, it would be clinically important if the study could be confirmed in a population-based cohort. Our team has already paid attention to this issue, and we hope to recruit a sufficiently large population for in-depth validation. Finally, our results suggest that SR may aggravate DCM mitochondrial damage through the PRDM16-PGC-1α pathway, thereby promoting apoptosis, fibrotic remodeling, leading to the development of a new type of mitochondria in DCM. Fibrotic remodeling led to deterioration of cardiac function and increased mortality. However, this study did not validate the rescue against PRDM 16. This is the main direction of our next research, and we are now screening the active ingredients of Chinese medicines isolated and extracted from our laboratory for those that can act on PRDM 16 for subsequent therapeutic studies.

## Materials and methods

### Animal procedure and tissue collection

Twenty 8-week-old KK–Ay mice (genetic type 2 diabetes model) and C57BL/6J mice were purchased from Beijing HFK Bioscience Co, Ltd (Beijing, China). All animal procedures were performed in accordance with the National Institutes of Health guide for the care and use of Laboratory animals (NIH Publications No. 8023, revised 1978) and by the Welfare and Ethics Committee of the Institute of Biological and Medical Engineering, Guangdong Academy of Sciences (Ethics Committee approval code: K2022002041118). Mice were housed in polycarbonate cages (5 per cage) at standard 12/12 day-night cycles, and water and food were provided ad libitum. The type 2 diabetes model mice were fed a high-fat diet, and the energy ratio of the high-fat diet was 60% from fat, 20% from protein, and 20% from carbohydrate.

Blood glucose was measured daily. KK mice with blood glucose concentrations above 15 mM (200 mg/dL) for two consecutive weeks were used for the next experiments [44]. Subsequently, the animals were randomized into 4 groups (10 animals per group). The groups were as follows (1) WT group: healthy C57BL/6J mice without any treatment; (2) WT + SR group: healthy C57BL/6J mice with 21 days of sleep restriction; (3) KK group: type 2 diabetic KK–Ay mice without any treatment; (4) KK + SR group: type 2 diabetic KK–Ay mice with 21 days of sleep restriction.

All animals were euthanized within 24 h of the end of the experiment. Mice were weighed, anesthetized by intraperitoneal injection of 1% sodium pentobarbital (0.2 mL/20 g body weight), and blood was taken by heart puncture from the right ventricle. The right auricle was clipped, and saline (50 mL/20 g body weight) was immediately infused into the left ventricle through an intravenous infusion device until the limbs turned white. Finally, heart samples were quickly removed and left ventricular tissue was dissected on ice, immediately placed in liquid nitrogen, and stored at −80 °C until use.

### Sleep restriction

The method of SR was adapted from the multiple platform method. Five mice were placed in a water tank (41 × 34 × 16.5 cm) with 13 platforms (3 cm in diameter), surrounded by water up to 1 cm beneath the surface. In this method, the animals were able to move within the tanks, jumping from one platform to another, freely maintaining diet and water. All controls were housed in controlled home cages and allowed to sleep freely on standard rodent food. The SR group was sleep-restricted for 3 weeks, 18 h daily [45–47]. After each 18 h of SR, the mice were allowed to sleep for 6 h (with sleep opportunity starting at 12 a.m.).

### HE staining, WGA staining, PAS staining and Masson staining

Cardiac tissues were fixed in 4% paraformaldehyde at 4 °C overnight, then dehydrated and paraffin-embedded. Paraffin sections (3 μm) were baked in an oven at 60 °C for 1.5 h and then processed for HE, Masson or PAS staining according to the manufacturer’s instructions. The morphological changes of the cardiac tissues were captured on camera with an optical microscope (Olympus, Tokyo, Japan). Sections were steamed in citrate antigen extract for 30 min and then incubated in WGA for 1 h. Nuclei were visualized by DAPI and photographed with a fluorescence microscope (Carl Zeiss LSM 880, Germany). Cardiomyocyte size and relative positive areas for PAS and Masson staining were determined using the ImageJ Quantitative Analysis System (Image Pro-Plus version 6.0). Five fields of view were randomly captured in each of the three sections that were used for each of these stainings in each group of five mice.

### Evaluation of echocardiography

Cardiac functional and structural parameters were measured in mice using a Vevo 2100 echocardiography system (Visualsonics, Toronto, Canada) equipped with a 30 MHz probe. Two-dimensional M-mode echocardiographic tracings were performed from the short-axis of the left ventricle to obtain averaged values of cardiac ejection fraction (EF), shortening fraction (FS), left ventricular diameters in systole and diastole (LVIDs and LVIDd), and left ventricular volumes in systole and diastole (LV Vols and LV Vold) from six successive cardiac cycles. Percent fractional shortening [%FS = (LVIDd- LVIDs)/ LVIDd × 100] and percent ejection fraction [%EF = (LV Vold-LV Vols)/LV Vold × 100] were utilized as indicators of cardiac systolic function.

### Real-time quantitative PCR (RT-qPCR)

Total RNA was extracted from tissues using TRIzol (Invitrogen). mRNA was reverse-transcribed to complementary DNA (cDNA) using a cDNA synthesis kit (Vazyme, China) according to the manufacturer’s instructions. RT-qPCR was performed using ChamQ SYBR qPCR Master Mix (Vazyme, China). Gene expression was quantified according to the RNA expression level of β-actin. The relative mRNA amounts of target genes were calculated by utilizing the 2^−ΔΔCt^ method. Primer sequences shown in chart below.

**Table:** 

Primer	Squence (5ʹ-3ʹ)
mmu-Col1a1-F	TAAGGGTCCCCAATGGTGAGA
mmu-Col1a1-R	GGGTCCCTCGACTCCTACAT
mmu-Tgfb1-F	CTCCCGTGGCTTCTAGTGC
mmu-Tgfb1-R	GCCTTAGTTTGGACAGGATCTG
mmu-ANP-F	GTGCGGTGTCCAACACAGAT
mmu-ANP-R	TCCAATCCTGTCAATCCTACCC
mmu-BNP-F	GAGGTCACTCCTATCCTCTGG
mmu-BNP-R	GCCATTTCCTCCGACTTTTCTC
mmu-Bax-F	AGACAGGGGCCTTTTTGCTAC
mmu-Bax-R	AATTCGCCGGAGACACTCG
mmu-Bcl2-F	GTCGCTACCGTCGTGACTTC
mmu-Bcl2-R	CAGACATGCACCTACCCAGC
mmu-PRDM16-F	TGCTGACGGATACAGAGGTGT
mmu-PRDM16-R	CCACGCAGAACTTCTCGCTAC
mmu-PGC-1α-F	TATGGAGTGACATAGAGTGTGCT
mmu-PGC-1α-R	CCACTTCAATCCACCCAGAAAG
mmu-IL1b-F	GCAACTGTTCCTGAACTCAACT
mmu-IL1b-R	ATCTTTTGGGGTCCGTCAACT
mmu-IL6-F	TAGTCCTTCCTACCCCAATTTCC
mmu-IL6-R	TTGGTCCTTAGCCACTCCTTC
mmu-TNFα-F	CCTGTAGCCCACGTCGTAG
mmu-TNFα-R	GGGAGTAGACAAGGTACAACCC
mmu-β-actin-F	CTAAGGCCAACCGTGAAAAG
mmu-β-actin-R	ACCAGAGGCATACAGGGACA

### Immunohistochemical analysis

The cardiac paraffin sections were deparaffinized and the deparaffinized sections were autoclaved in 10 mM sodium citrate (pH 6.0) solution for antigen repair. Sections were then incubated in 3% hydrogen peroxide to block endogenous peroxidase activity and incubated with primary antibodies (mouse monoclonal antibodies against Collagen I and α-SMA; Abcam) for 2 h at room temperature. Sections were rinsed 3 times with PBS and then incubated with the corresponding secondary antibody for 2 h at room temperature. Subsequently, the sections are rinsed 3 times with PBS and incubated in 0.02% diaminobenzidine solution for 2–8 min. After restaining with hematoxylin, the sections were washed briefly, sealed with resin and photographed under an optical microscope (Olympus, Tokyo, Japan). The relative area of positive Collagen I and α-SMA staining were determined by using Image Pro-Plus. Five fields of view were randomly acquired in each of three sections from each group of five mice.

### TdT-mediated dUTP nick end-labeling (TUNEL) staining

The TUNEL staining kit (In Situ Cell Death Detection Kit, TMR red, Roche) was used to detect and quantify cell death, and the experiments were performed following the manufacturer’s instructions. Carl Zeiss LSM 880 fluorescence microscope (Germany) was used to capture the images. Quantification was analyzed with the Image-Pro Plus 6.0 software.

### Transmission electron microscopy (TEM)

The mice’s hearts were harvested and cut into 1 mm^3^ section from the apical of the left ventricle. They were then fixed in 2.5% Glutaraldehyde for 1 h, then incubated at 4 °C overnight. The heart samples were then post-fixed and filmed, and the images were collected using a HITACHI HT7700 electron microscopy at 80 kV (Hitachi, Tokyo, Japan). Magnification of ×5000 and ×15,000 were captured, and the mitochondrial morphology were assessed. Mitochondria density and mitochondria with cristae loss were manually traced and quantified (*n* = 10–15 images/heart) for each sample using ImageJ.

### Biochemical parameters measurement

T-AOC, SOD, MDA, IL-6, IL-1β and TNF-α were measured in plasma according to the kits provided by the manufacturer. All assay kits were purchased from the Nanjing Jiancheng Bioengineering Institute (Nanjing, China). ATP (E-BC-K157-M) and ROS (E-BC-K138-F) in myocardial tissue were assayed using kits from Elabscience (Wuhan, China).

### Cell cultures and in vitro transfection

The experimental protocol was performed as previously described in detail [48]. The cell line H9C2 was cultured in DMEM medium with 10% fetal bovine serum at 37 °C in 5% CO2 and atmospheric oxygen. The siRNA sequences were: siPRDM16-1 sense strand, 5′-CCAUACAGGUGCAAGUACUGU-3′; siPRDM16-1 antisense strand, 5′-AGUACUUGCACCUGUAUGGCU-3′; siPRDM16-2 sense strand, 5′-GGACAGUGACAGAGACAAATT-3′; siPRDM16-2 antisense strand, 5′-UUUGUCUCUGUCACUGUCCTT-3′; siPRDM16-3 sense strand, 5′-GGACGCAGAUCAAGUGCAATT-3′; siPRDM16-3 antisense strand, 5′-UUGCACUUGAUCUGCGUCCTT-3′; siNC sense strand, 5′- UUCUCCGAACGUGUCACGUTT-3′; siNC antisense strand, 5′-ACGUGACACGUUCGGAGAATT-3′. We tested the efficiency of these three siRNAs in knocking down PRDM16 expression, and the results showed that all three siRNAs could effectively interfere with PRDM16 expression, and the most efficient sequence siPRDM16-1 was used in this study. Cells seeded in 6-well plates were grown to 70–80% before transfection. Lipofectamine 3000 (Invitrogen, Carlsbad, CA) reagent and 20 μM siRNA were mixed with Opti-MEM medium (Invitrogen, Carlsbad, CA) were incubated for 20 min at room temperature and then incubated with cells for 4–6 h. Thereafter, the cells were incubated with regular medium instead for 48 h.

### Western blotting

Myocardial tissue was thoroughly ground with a tissue grinder, then lysed by adding RIPA buffer containing protease and phosphatase inhibitors for 30 min at 4 °C. Protein samples were separated on 12% sodium dodecyl sulfate polyacrylamide gels (SDS-PAGE). The gel was transferred onto a polyvinylidene difluoride (PVDF) membrane (Millipore, Billerica, MA, USA). The membranes were blocked with 5% skimmed milk for 1 h at room temperature, incubated with primary antibody overnight at 4 °C, and then incubated with horseradish peroxidase (HRP)-conjugated secondary antibody for 1 h. Protein band intensities were quantified using Image J software and normalised with β-actin. Primary antibodies were as follows: anti-PRDM16 antibody (1:1000 dilution, ab303534, Abcam, Cambridge, UK); anti-PGC-1α antibody (1:1000 dilution, ab313559, Abcam, Cambridge, UK) and anti-β-actin antibody (1:1000 dilution, #4970, Cell Signaling Technology, Danvers, MA, USA).

### Cellular mitochondrial reactive oxygen species and membrane potential detection

Cellular ROS was measured using Reactive Oxygen Species Assay Kit (S0033S, Beyotime, China) and mitochondrial membrane potential was determined using a TMRE mitochondrial membrane potential assay kit (C2001S, Beyotime, China) according to the manufacturer’s protocol.

### Statistical analysis

The animal assays involved were independently repeated for at least six mice. Data from replications were averaged and presented as the mean ± SEM. GraphPad Prism 9 (GraphPad, CA, USA) was used for all statistical analyses. Student’s *t*-test was used to compare two groups. Group differences were tested using Two-way ANOVA followed by Bonferroni’s *post hoc* test. *P* values < 0.05 were considered to indicate statistically significant differences. All experiments were performed in triplicate at a minimum.

## Supplementary information

Original western blots

## Data Availability

The authors declare that all data supporting the findings of this study are available within the article.
