# Supplementary material for: Sleep restriction exacerbates cardiac dysfunction in diabetic mice by causing cardiomyocyte death and fibrosis through mitochondrial damage
Source: Cell Death Discov. 2024 Oct 21;10:446. doi: 10.1038/s41420-024-02214-w (PMC11494183; doi:10.1038/s41420-024-02214-w)

**The original files of the western blots**

The original files of the western blots, Related to Figure 7.

PRDM16


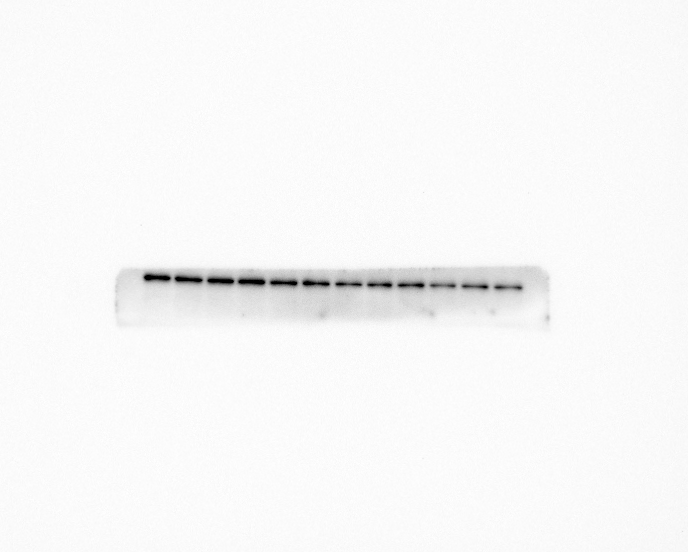


PGC-1α


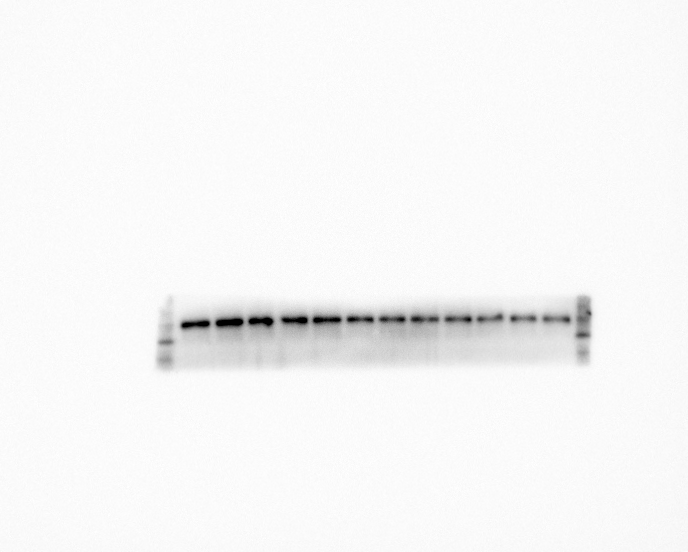


β-actin


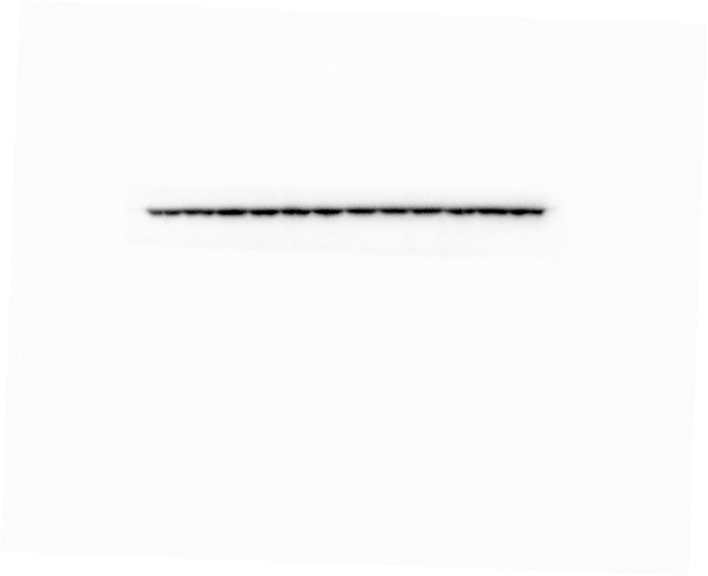


PRDM16


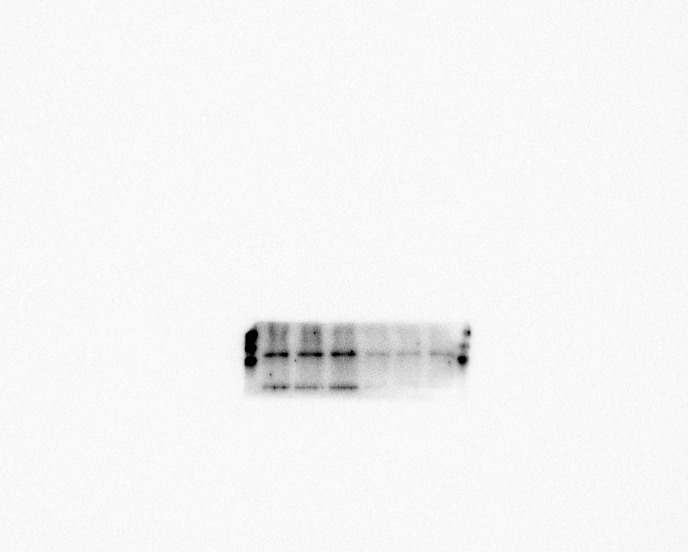


β-actin


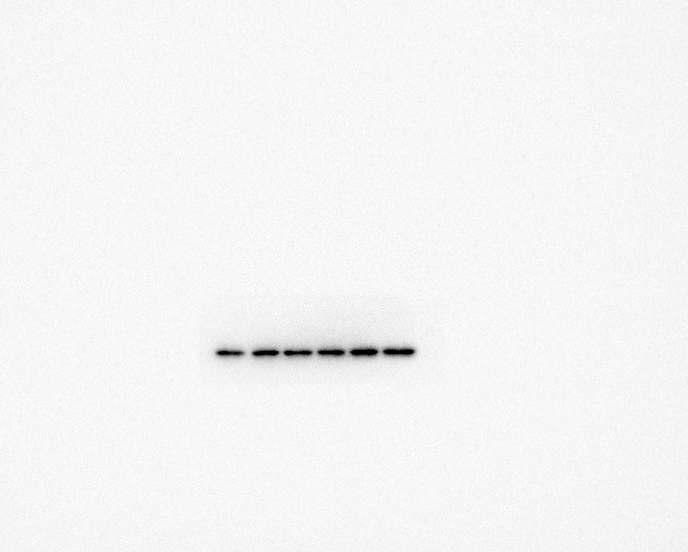

Supplement: Supplementary file 1 — Original western blots [file 41420_2024_2214_MOESM1_ESM.docx]
